# Acoustic Enhancement of Sleep Slow Oscillations and Concomitant Memory Improvement in Older Adults

**DOI:** 10.3389/fnhum.2017.00109

**Published:** 2017-03-08

**Authors:** Nelly A. Papalambros, Giovanni Santostasi, Roneil G. Malkani, Rosemary Braun, Sandra Weintraub, Ken A. Paller, Phyllis C. Zee

**Affiliations:** ^1^Center for Circadian and Sleep Medicine, Department of Neurology, Feinberg School of Medicine, Northwestern University, ChicagoIL, USA; ^2^Biostatistics Division, Feinberg School of Medicine, Northwestern University, ChicagoIL, USA; ^3^Department of Engineering Sciences and Applied Mathematics, Northwestern University, EvanstonIL, USA; ^4^Cognitive Neurology and Alzheimer’s Disease Center and Department of Psychiatry and Behavioral Sciences, Northwestern University, ChicagoIL, USA; ^5^Department of Psychology, Northwestern University, EvanstonIL, USA

**Keywords:** acoustic stimulation, sleep, aging, slow waves, memory

## Abstract

Acoustic stimulation methods applied during sleep in young adults can increase slow wave activity (SWA) and improve sleep-dependent memory retention. It is unknown whether this approach enhances SWA and memory in older adults, who generally have reduced SWA compared to younger adults. Additionally, older adults are at risk for age-related cognitive impairment and therefore may benefit from non-invasive interventions. The aim of this study was to determine if acoustic stimulation can increase SWA and improve declarative memory in healthy older adults. Thirteen participants 60–84 years old completed one night of acoustic stimulation and one night of sham stimulation in random order. During sleep, a real-time algorithm using an adaptive phase-locked loop modeled the phase of endogenous slow waves in midline frontopolar electroencephalographic recordings. Pulses of pink noise were delivered when the upstate of the slow wave was predicted. Each interval of five pulses (“ON interval”) was followed by a pause of approximately equal length (“OFF interval”). SWA during the entire sleep period was similar between stimulation and sham conditions, whereas SWA and spindle activity were increased during ON intervals compared to matched periods during the sham night. The increases in SWA and spindle activity were sustained across almost the entire five-pulse ON interval compared to matched sham periods. Verbal paired-associate memory was tested before and after sleep. Overnight improvement in word recall was significantly greater with acoustic stimulation compared to sham and was correlated with changes in SWA between ON and OFF intervals. Using the phase-locked-loop method to precisely target acoustic stimulation to the upstate of sleep slow oscillations, we were able to enhance SWA and improve sleep-dependent memory storage in older adults, which strengthens the theoretical link between sleep and age-related memory integrity.

## Introduction

As the population of adults over the age of 65 continues to increase, it is critical to further elucidate the relationships between sleep and cognitive function. Age-related cognitive decline can be seen across multiple cognitive domains such as executive function, processing speed, and memory (Schaie et al., 1998). A decline in hippocampal-dependent declarative memory, or the ability to consciously recall facts and episodic knowledge, is a frequent complaint of older adults (Newson and Kemps, 2006) and a potential precursor to dementia (Hohman et al., 2011). Age-related decline in declarative memory typically has been attributed to failures in encoding and/or retrieval of information (Luo and Craik, 2008), whereas consolidation has been under-emphasized. Although age is a significant risk factor for memory loss and dementia, there may be modifiable factors that also contribute to memory decline, such as sleep.

Deep sleep, also known as slow wave sleep (SWS), may be particularly relevant for understanding the intersection of sleep, aging, and memory. SWS is characterized by slow waves in the delta frequency band (0.5–4 Hz) of at least 75 μV. Slow wave activity (SWA), a quantitative physiologic measure of SWS, is the electroencephalographic (EEG) power in the delta frequency range. In young individuals, sleep has been shown to play an important role in memory consolidation, and sleep deprivation can disrupt the ability to encode and consolidate new memories (Rasch and Born, 2013). SWS appears to be particularly conducive to hippocampal-dependent memory consolidation (Plihal and Born, 1997; Born et al., 2006; Marshall and Born, 2007). Furthermore, selective suppression of sleep slow waves leads to poor memory encoding (Van Der Werf et al., 2011) and visuomotor learning (Landsness et al., 2009). The theory of active system consolidation of declarative memories suggests that slow oscillations drive repeated reactivation of memory traces in the hippocampus (Born and Wilhelm, 2012; Rasch and Born, 2013). Consolidation, which results in strengthening of cortical representations and decreased dependence on the hippocampus for retrieval (Paller, 2009), may be facilitated when the activity of thalamo-cortical network is synchronized via slow oscillations (Diekelmann et al., 2009). In addition to SWS, Rapid Eye Movement (REM) sleep may also contribute to consolidation of episodic (Rauchs et al., 2004) and procedural memories (Plihal and Born, 1997). At any rate, a key question is whether specific changes in sleep contribute to memory impairment in aging populations.

Sleep in older adults is characterized by frequent awakenings and a prominent reduction in REM, SWS, and SWA (Ohayon et al., 2004; Edwards et al., 2010). Although word pair recall in older adults has been associated with duration of non-REM/REM sleep cycles (Mazzoni et al., 1999), REM sleep deprivation has been shown to have no effect on memory consolidation (Hornung et al., 2007). Much is unknown about the specific mechanisms of age-related changes in sleep physiology, but recent evidence suggests that gray-matter atrophy in the medial prefrontal cortex underlies age-related decline in SWA (Mander et al., 2013b). SWA has indeed been shown to be associated with declarative memory performance in older adults (Westerberg et al., 2012; Mander et al., 2013b). Given this evidence implicating SWA, it is biologically plausible that memory storage can be enhanced in older adults by promoting slow wave synchronization during sleep.

Manipulation of SWS provides a powerful tool both to investigate causal relationships between sleep and memory, and to improve memory consolidation. Whereas slow-oscillatory electrical stimulation can increase SWA and boost memory in older adults (Westerberg et al., 2015), this methodology has practical limitations, due to setup complexity and potential safety issues that would impede long-term use. In contrast, acoustic stimulation has distinct advantages, such as feasibility for repeated at-home use and individualized adjustments that could be automated in real-time. In addition, SWA during stimulation can readily be analyzed, which is not the case for electrical stimulation. Pulses of pink noise targeted to the upstate of intrinsically generated slow waves increased SWA and improved word pair recall in young adults (Tononi et al., 2010; Ngo et al., 2013b; Ong et al., 2016), but such studies using acoustic stimulation in older adults are lacking.

The goal of the present study was to determine whether acoustic stimulation in sleep can boost SWA and improve memory in older adults. We developed an automated, adaptive algorithm that can monitor the endogenous slow oscillatory activity in the EEG and phase-lock the timing of acoustic stimuli to a desired phase of the slow wave (Santostasi et al., 2016). This phase-locked loop (PLL) has been previously utilized to deliver intervals of acoustic pulses to the upstate of the slow wave during SWS that resulted in an increase in SWA in young adults during daytime naps (Ong et al., 2016; Santostasi et al., 2016). However, the feasibility of this method has not been tested in older adults. We examined changes in memory using a randomized crossover design comparing one night of acoustic stimulation to one night of sham stimulation; in both conditions, participants completed a declarative memory test before and after sleep.

## Materials and Methods

### Participants and Experimental Design

Thirteen cognitively healthy older adults [mean age 75.2 years (range 60–84); three men; mean education 17.5 years (range 16–24)] were recruited from the Northwestern University Cognitive Neurology and Alzheimer’s Disease Center Clinical Core registry. All participants underwent a research neuropsychological and neurological evaluation according to standardized data collection procedures of the Uniform Data Set (Morris et al., 2006; Weintraub et al., 2009) and were determined to be cognitively normal for age. Exclusion criteria for study participation included history of neurological disease, unstable medical or psychiatric conditions, alcohol or substance abuse, history of seizures, history of a sleep disorder, or hypnotic or psychoactive drug use. Four additional participants were not included in the final analysis due to short SWS duration (<3 min), usage of a hearing aid, or excessive EEG artifact. The Northwestern University Institutional Review Board approved this study. Written informed consent was obtained from all participants prior to study participation.

Participants completed two overnight visits, at least 1 week apart (mean 13.2 days). Acoustic stimulation was delivered during one of the overnight sessions (STIM) and sham stimulation during the other (SHAM). Sham stimulation consisted of an identical setup with PLL tracking, but no sounds were delivered. The order of the visits was randomized, resulting in six participants receiving acoustic stimulation and seven participants receiving sham stimulation on the first visit. Participants were blinded to the condition at each visit. Participants arrived at the sleep laboratory at 18:00. Participants completed a word pair learning task and a cued-recall test (see below) 90 min prior to their self-reported bedtime. Participants were fitted with soft headphones specifically designed for sleep (Acoustic Sheep Sleep Phones, SP4BM). Prior to sleep, the intensity of the stimulation was adjusted to a level that the participant considered acceptable and would not disturb them during sleep (30–50 dB). Lights were turned off at self-reported bedtime from screening questionnaires, and participants were given at least 8 h of opportunity to sleep during which polysomnography was performed. One hour after waking, participants completed the delayed cued-recall test. To examine any differences in subjective feelings of sleep quality and alertness, participants completed mood, alertness, and sleep-quality questionnaires 45 min after waking.

### Polysomnography Recording

Electroencephalographic was recorded from nine channels (international 10–20 system: Fpz, F3, F4, C3, C4, P3, P4, O1, O2) referenced to left mastoid (V-Amp amplifier, Brain Products GmbH). Impedances were lowered to 10 kΩ. Electro-oculogram (EOG) was recorded using two electrodes placed lateral to each eye and chin electromyogram (EMG) was recorded using three chin electrodes. The full EEG data set was collected using Brain Vision Recorder software (Brain Products GmBH) with a 500 Hz sampling frequency and stored unfiltered for off-line analysis and sleep scoring. Prior to sleep scoring, raw EEG data were band-pass filtered with cut-off frequencies at 0.5 and 35 Hz. In order to control stimulation during recording, signal from one channel (Fpz) was also passed to a Matlab Application Programming Interface (API) and was further pre-processed online by applying a band-pass filter (Chebyshev second order, 0.05 dB pass band ripple) with cut-off frequencies at 0.5 and 38 Hz. Data were down sampled to 100 Hz to avoid aliasing and were stored offline separately. Primary analyses used the Fpz data from Matlab unless stated otherwise.

### Phase-Locked Acoustic Stimulation

A Matlab script (R2014b, MathWorks, Natick, MA, USA) was used for online detection of slow waves at channel Fpz and to control acoustic stimulation in a phase-locked manner as described previously (Santostasi et al., 2016). Channel Fpz was used as the detection site because this channel can most easily be used by individuals for at-home recording, which is a future goal for this procedure. Also, slow wave amplitudes appear high in this general region of the scalp (Massimini, 2004; Tononi et al., 2010; Nir et al., 2011), so the algorithm can readily track and lock to the slow oscillation. The algorithm used a simplified method of detecting slow waves by identifying relative average root mean square (RMS) in the alpha, beta, and delta EEG frequency bands recorded from Fpz. The delta RMS was calculated as a moving average of the previous 10 s of EEG activity. Stimulation does not begin until two criteria are met. First, average delta RMS must remain above 10 μV for at least 75 s. Second, the algorithm must identify slow waves with the negative half-wave amplitude exceeding -40 μV and lasting between 0.25–2 s, and there must be six slow waves within a 30 s period. When this occurred, the PLL began oscillating at a central frequency of 0.85 Hz and continuously adjusted its frequency to phase-lock to the endogenous slow oscillations (bandwidth 3.7 Hz). The advantages of this method of acoustic stimulation for detecting and targeting slow waves have been described (Santostasi et al., 2016), but it is important to note that the ability of the PLL to frequency match anywhere within the delta range of 0.5–4 Hz makes this method uniquely suited to target the variable frequency and amplitude of slow waves in an older population.

**FIGURE 1:**
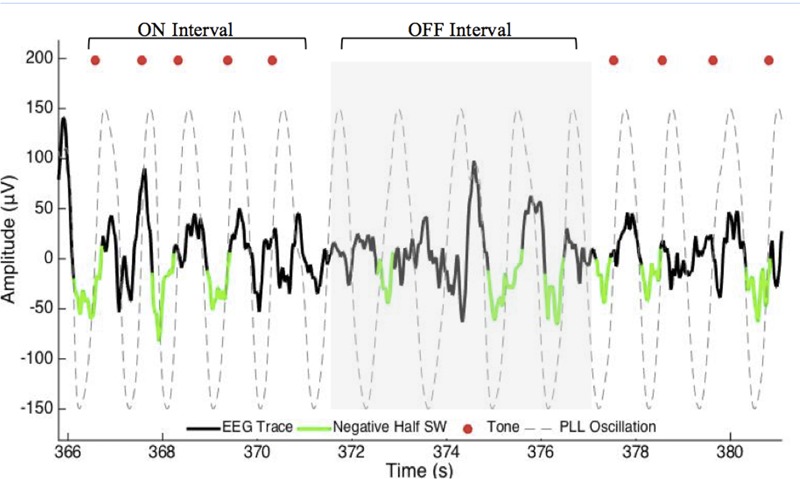
**Excerpt of data for phase-locked loop (PLL) pulse delivery for ON and OFF intervals.** The PLL continuously models phase (gray-dotted line) to target the upstate of the slow wave. The acoustic stimulation is delivered in blocks of five pulses ∼1.2 s apart (red dots, ON Interval) followed by an off period of ∼6 s (OFF Interval). PLL, Phase-locked loop; SW, slow wave.

**FIGURE 2:**
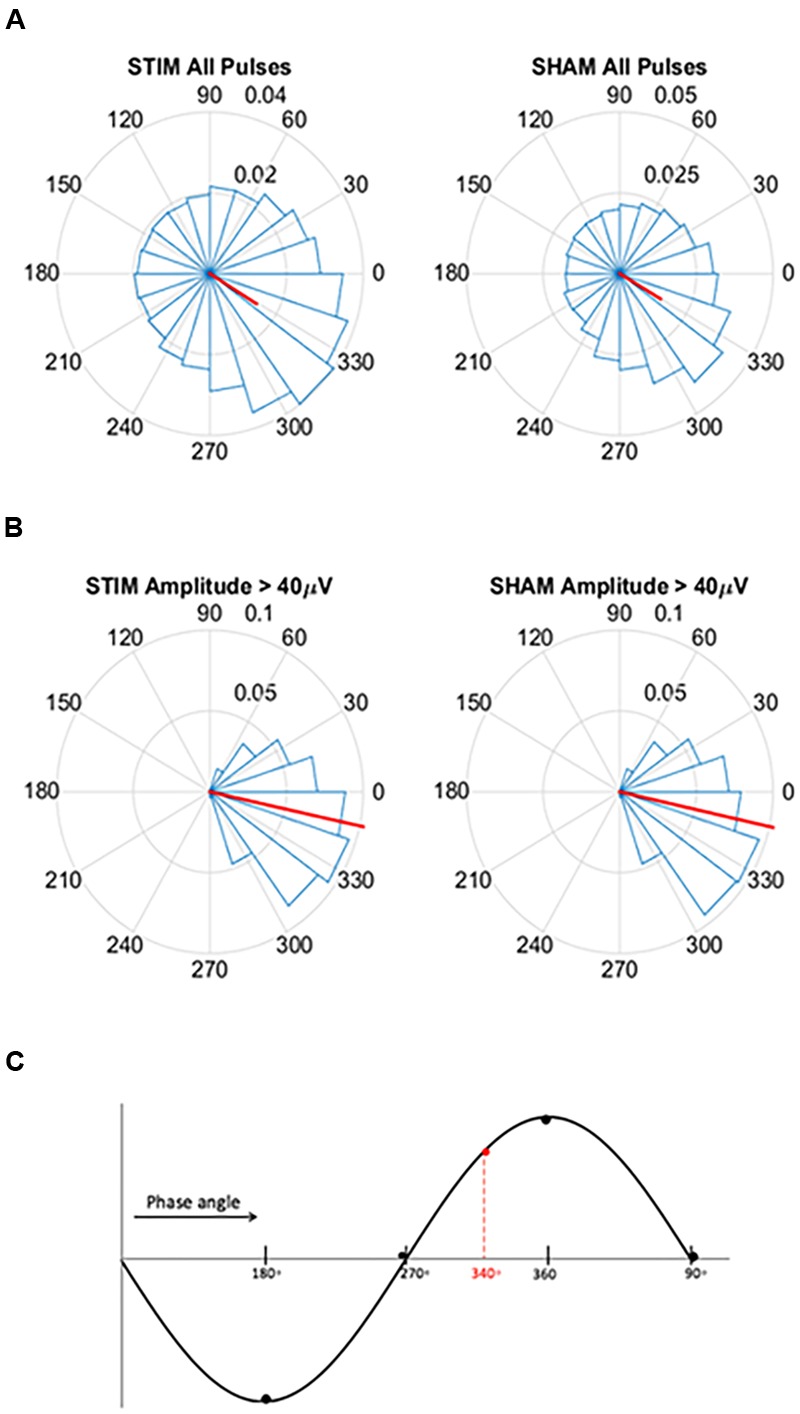
**Circular histograms for STIM and SHAM conditions averaged across all participants. (A)** Phase spread of all pulses, including pulses that inadvertently hit other phases. Red lines indicate mean phase vector and spread is depicted in 20 bins of 18 degrees. During the SHAM night, time of pulses was marked, but no sound was played. **(B)** Phase spread of all pulses for slow waves with amplitudes greater than 40 μV for both the STIM and SHAM. **(C)** Graphical illustration of phase angle, dotted red line indicates location of target phase on the slow wave.

The acoustic stimulation routine consisted of 50 ms pulses of pink (1/f) noise delivered in blocks of 5 (ON interval). Each of the five pulses of the ON interval was separated by an average of 1.2 s. Each ON interval was followed by a pause of five PLL oscillations (∼6 s) detected in the same way (OFF interval). Pulse delivery was targeted 20 degrees prior to the peak of the upstate of the slow wave (**Figures 1, 2C**). This target phase accounted for hardware delays of 60 ms (data packet delivery and sound card activation). The acoustic stimulation routine continued unless an arousal or change in sleep stage was detected, indicated by an increase in alpha or beta RMS (8 and 21 μV) for more than 1 s, in which case a 30 s pause was initiated.

### Memory Task and Subjective Sleep

A verbal paired-associate learning task was used to assess declarative memory before and after sleep. The task began 90 min prior to lights out. During the learning phase participants viewed 88 moderately associated word pairs (e.g., energy-oil) as used in previous studies on sleep and memory (Marshall et al., 2004; Westerberg et al., 2012, 2015; Ngo et al., 2013b, 2015). The word pairs were presented once, centrally on a computer screen, for 4 s each with an inter-stimulus interval (ISI) of 1 s. Participants were instructed to memorize each pair. Immediately following the learning phase, participants completed a cued-recall test in which they were presented with the first word of each pair, in random order, and were given unlimited time to recall the matching word or to state “don’t know.” Participants were encouraged to guess if they thought they might have the answer. Immediately following a recall attempt, the correct word pair was displayed on the screen for 1 s. In the morning, 1 h after waking, participants completed a second cued-recall test with feedback of the correct word. For the morning test, words were presented in a different random order. A different learning word list was used for each overnight visit. Memory performance was measured as both a raw change in word recall and as a percent change from the evening score: [(morning score – evening score)/evening score × 100]. To assess subjective mood, attention, and sleep quality, participants completed the Karolinska Sleep Diary and the Global Vigor Affect Visual Analog Scale 45 min after waking.

### Sleep and Spectral Analysis

To examine differences in sleep structure between STIM and SHAM conditions, an experienced rater (RM) blinded to the experimental condition (STIM/SHAM) did offline scoring of sleep with Polysmith reading software (v.8.0, Nihon Kohden) using the AASM scoring criteria. Total sleep time, time spent in each stage of sleep (N1, N2, N3, REM sleep), and arousal index (number per hour) for the stimulation and sham night were then calculated (Iber et al., 2007).

Auditory event-related potentials (ERPs) were examined to assess the effects of stimulation. EEG data were analyzed in a 6 s window, time-locked to the first pulse of the ON intervals. The pre-stimulus time interval preceding the start of each ON interval (time = 0) was the later portion of the prior OFF interval. To elucidate whether response to stimulation diminished across the ON intervals, ERPs were computed separately for pulse one through five, each with a 2 s window (0.5 s before the pulse, and 1.5 s after). The average peak-to-peak amplitude within the time window was calculated for (1) all potentials and (2) slow waves with peak-to-peak amplitude ≥75 μV. For all potentials, the peak-to-peak amplitude was considered to be the largest negative and largest positive consecutive peaks. The peak-to-peak amplitudes of the slow waves were calculated by first identifying the slow wave within the time window using previously published methods (Mölle et al., 2002, 2011). Each positive-to-negative zero crossing was identified and any two consecutive positive-to-negative zero crossings between 0.25 and 1.5 s were evaluated. For intervals meeting this criteria, peak-to-peak amplitude was considered to be the largest negative peak to the largest positive peak. Only slow waves with a peak-to-peak amplitude ≥75 μV were included in the analysis. Amplitudes were averaged across all time windows following ON and OFF intervals of each night and then averaged across participants.

Spectral analysis focused on changes in spectral content in response to acoustic stimulation within the ON and OFF intervals of the stimulation period, with particular focus on SWA defined as delta power (0.5–4 Hz). All spectral analysis was performed in Matlab, on channel Fpz, with custom-made scripts and functions from the EEGlab toolbox (Delorme and Makeig, 2004, RRID:SCR_007292). EEG data from the stored Matlab file were band-pass-filtered between 0.25–25 Hz and averaged across all the ON intervals and OFF intervals separately for each night. A Fast Fourier Transform (4 s window, 50% overlap) was used to identify mean power spectral density in the SWA, theta (>4–8 Hz), alpha (>8–12 Hz), slow spindle (>11–13 Hz), fast spindle (>1315 Hz), and beta (>1620 Hz) frequency bands for the ON and OFF intervals of the STIM and SHAM nights. First, we examined average power for SWA in NREM sleep and in ON and OFF intervals. Then, to account for differences between nights, average power for each frequency band was normalized to the total power in ON and OFF intervals for that specific frequency. Average percent power change from the SHAM ON intervals to the STIM ON intervals was calculated.

Event-related spectral perturbations (ERSPs) were created to quantify time-varying changes in the spectral content between the STIM ON intervals and the SHAM ON intervals. As in the ERPs, the ON intervals were defined as the 6 s window time-locked to the first pulse of the ON interval. The EEGLAB function ‘newtimef,’ with Morlet wavelet cycles of 0.3 cycles at 0.2 Hz to 25.6 cycles at 26 Hz, was used to compute spectral estimates across each ON interval, which was normalized to a pre-stimulus baseline (beginning 200 ms prior to the first pulse of each ON interval). The ratio of the output in decibels (dB) from the ‘newtimef’ function for the STIM and SHAM was calculated [power ratio = 10^(STIM/10)^/10^(SHAM/10)^] and was then expressed in dB [ratio_dB_ = 10 * log_10_ (power ratio)]. This computation was performed separately for each subject. Finally, an average was taken across all subjects to reflect the average ERSP in response to STIM relative to SHAM.

Spindle analysis was performed for the duration of PLL activation (all ON and OFF intervals) to assess the modulation of spindles by acoustic stimulation. Spindle detection was done as described by Ferrarelli et al. (2007) using the Hilbert transform to calculate spindle amplitude envelopes in channel Fpz. Fpz was of interest given findings that prefrontal spindle generation is related to hippocampal dependent memory in older adults. Data was filtered between 12–16 Hz and spindles were identified based on mean spindle envelope and timing criteria as follows. The spindle envelope had to be larger than the difference between the lower and upper thresholds, set at 1 and 2.5 standard deviations above the mean envelope, respectively. Spindle duration was required to be at least 300 ms and at most 2500 ms. Spindle duration limits were adjusted to capture spindles >300 ms that appear in N3 sleep in older adults (Warby et al., 2014) and that 99% of spindles are <2500 ms (Warby et al., 2014; Rosinvil et al., 2015). Two parameters were investigated: spindle amplitude and density. Spindle density was calculated as the number of spindles per minute of PLL activity.

To assess the ability of the PLL to target the positive half-wave in this older population we created circular histograms for the average phase in which each pulse was delivered. The target phase of pulse delivery was 340 degrees, where 360 degrees represents the peak of the upstate. First we used a zero-phase digital filter with transfer function coefficients of a second order bandpass Butterworth filter in the delta band. We identified the phase angle at each pulse delivery and Hilbert transformed the EEG signal to identify the instantaneous phase at each pulse delivery. In our phase analysis, we initially investigated the phase targeting taking into account all pulses, and then we only took into account slow waves with amplitude greater than 40 μV. For both conditions, we averaged the instantaneous phase for all slow waves and then those with amplitudes greater than 40 μV. Circular histograms were created with 20 bins of 18 degrees and a mean vector. Finally, to determine if memory performance was related to phase, we first correlated the two and then separated the percent improvement in memory recall between the STIM and SHAM conditions by above and below-median performance. Histogram counts were normalized to the total number of counts across all participants. We then calculated the average target phase of the two groups.

We tested the hypothesis that the relative increases in SWA observed during the STIM ON period to the STIM OFF period influenced the improvement in memory. First, the percent change in the normalized SWA between the ON and OFF intervals of the SHAM night was subtracted from the percent change in the normalized SWA of the ON and OFF intervals of the STIM night. Second, the percent improvement in evening to morning word pair recall for SHAM condition was subtracted from the percent improvement in evening to morning word pair recall for the STIM. Associations between these two measures (% change SWA, % change in memory) were examined. Additionally, we explored relationships between the change in fast spindle activity and change in memory, as evidence in younger adults suggest a causal relationship between the two (Ngo et al., 2013b).

### Statistical Analysis

SPSS v. 16.0 (RRID: SCR_002865), Matlab (RRID: SCR_001622), and R (RRID: SCR_001905) were used for statistical analysis. Within subject differences in sleep characteristics and memory outcomes were evaluated using paired *t*-tests or Wilcoxon signed-rank tests. Normality assumptions for pairwise differences were first checked using the ‘shapiro.test’ function in R, and paired *t*-tests were applied where the appropriate normality assumptions were met. In the case of spindle density, normality assumptions were violated (*p* = 0.038, Shapiro–Wilk test), so a non-parametric Wilcoxon signed rank test for paired data was applied. Peak-to-peak amplitudes for (1) all event-related potentials and (2) slow wave potentials for pulse one through five were evaluated using ANOVAs. To account for the repeated measures (peak-to-peak amplitude measured at five time points), the R lme4 package (Bates et al., 2015) was used to fit a mixed-effects model conditioned on the subject ID (random effect), with fixed effects Time (pulse 1–5), Condition (STIM/SHAM), State (ON/OFF), and the interactions between them. Contributions of the fixed effects were assessed using the ‘Anova’ function of the R car package (Fox and Weisberg, 2011) to compute Type-III *F*-tests. Phase targeting was evaluated using circular statistics from the CircStat toolbox (Berens, 2009). Spectral power differences as a function of frequency and time were assessed using paired *t-*tests across 64 frequency and 200 time interval bins, followed by FDR adjustment of the *p*-values (Yoav and Hochberg, 1995; Yoav and Yekutieli, 2001) to correct for the multiple comparisons. A two sample *t*-test was used to evaluate differences in phase targeting for the below and above-median memory performance groups. Associations were evaluated using Pearson correlations. A *p-*value <0.05 was considered significant. Mean and standard error of the mean (SEM) are reported unless otherwise noted.

## Results

### Phase-Locked Auditory Pulses Successfully Target Upstate of Slow Oscillation

For pulses across all slow waves, the mean instantaneous phase of pulse delivery was for STIM was 328.5 (*SD* = 73.4) degrees (**Figure 2A**). Pulse delivery phase computed in the same way for SHAM, when no sounds were actually presented, was 329.7 (73.1) degrees. For slow waves with amplitudes greater than 40 μV the mean instantaneous phase was 347.8 (32.8) degrees for STIM and 347.5 (32.0) degrees for the SHAM night (**Figure 2B**). These results indicate that larger slow waves result in better tracking performance of the PLL and thus better accuracy for the target phase as represented in **Figure 2C**.

### Auditory Event-Related Potentials Showed Persistent Response to Stimulation

**FIGURE 3:**
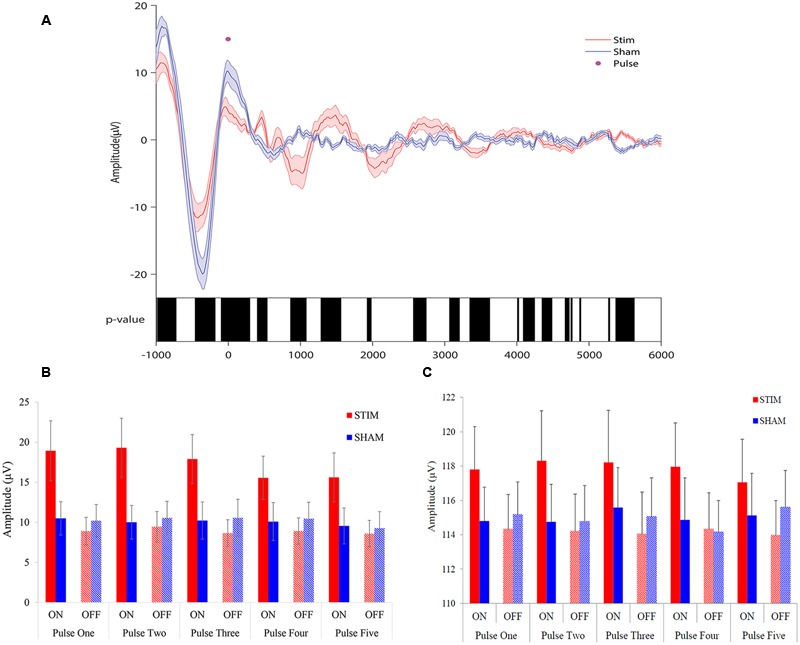
**Overlay of auditory ERPs for STIM and SHAM nights. (A)** Grand average ERPs for the STIM (red) and SHAM (blue) nights aligned to the first pulse of each ON interval (time = 0). Black bars indicate *p* < 0.05 between STIM and SHAM conditions. **(B)** Average peak-to-peak amplitude of 1.5 s ERPs windows, time locked to pulse one through five, for STIM and SHAM ON and OFF intervals. **(C)** Average slow-wave peak-to-peak amplitudes ≥75 μV for the 1.5 s ERP windows time locked to pulse one through five.

To observe the effects of stimulation, we first examined EEG signals time-locked to the first pulse of each ON interval. Acoustic pulses elicited an increase in amplitude of the slow wave during STIM ON intervals compared to SHAM ON intervals (**Figure 3A**). A negative potential observed about 500 ms prior to stimulus onset (time = 0) was smaller in amplitude for the STIM compared to the SHAM condition (**Figure 3A** black bars, *p* < 0.05), most likely indicating that the response to the prior stimulation period was followed by a refractory effect during the subsequent OFF interval. There was a tapering effect seen after the second oscillation that might be due to temporal jitter of slow waves across trials, increasing with lag from the first pulse. We therefore examined ERPs time-locked to each of the five pulses of the ON intervals for the STIM and SHAM conditions.

ANOVA results for peak-to-peak amplitudes of all potentials for the 1.5 s intervals, time-locked to pulse one through five (**Figure 3B**), indicated that there was a significant Condition × State interaction (*p* < 0.0001), without a significant Condition (STIM/SHAM, *p* = 0.13), State (ON/OFF, *p* = 0.87) or Time (pulse 1–5; *p* = 0.47) effect. When examining slow waves with peak-to-peak amplitudes ≥75 μV, ANOVA results similarly indicated a significant Condition × State interaction (*p* < 0.0001), without significant main effect contributions from Condition (STIM/SHAM; *p* = 0.13) or State (ON/OFF; *p* = 0.91) on peak-to-peak amplitude. There was no significant effect of Time (pulse 1–5; *p* = 0.97), suggesting that the peak-to-peak amplitude did not differ across time for large slow waves. Taken together, the results indicate that amplitude was significantly increased during ON compared to OFF periods during STIM (but not SHAM) intervention for all potentials (**Figure 3B**) and larger slow waves (**Figure 3C**).

### Spindle Density and Amplitude Increased while Overall Sleep Macrostructure Remained Largely Unchanged

Stimulation was delivered primarily during NREM. The criteria for stimulation in the STIM condition were achieved for 11.8 (10.9) min for N1, 30.0 (17.0) min for N2, 84.4 (10.2) min for N3, and minimally in other stages [REM: 2.3 (3.1) min, WAKE: 3.1 (2.3) min]. As shown in **Table 1**, total sleep time, time spent in stages N1, N2, and REM sleep, wake after sleep onset, and sleep efficiency did not differ between the STIM and SHAM nights. However, two non-significant trends were apparent; time spent in SWS was 12.5 min greater in the SHAM night, and sleep latency was 8.8 min longer in the STIM night. Arousal indices did not differ between nights. On the other hand, a stimulation-based increase in sleep spindles was statistically significant. Spindle density was 0.3 spindles/min higher and spindle amplitude was 1.2 μV higher with STIM.

**Table 1:** Mean (SEM) of the macrostructure of overnight sleep for the STIM and SHAM nights.

	SHAM	STIM	*p-*value
Total sleep time	366.2 (25.7)	369.5 (16.6)	0.88
Wake time	171.5 (33.7)	187.6 (18.4)	0.47
Stage N1	30.3 (6.7)	37 (8.3)	0.12
Stage N2	204.5 (21.7)	216.5 (17.8)	0.51
Stage N3	63.9 (12.0)	51.5 (12.0)	0.06
Stage REM	67.4 (10.8)	64.5 (8.0)	0.70
Sleep latency	17.7 (6.9)	26.6 (5.21)	0.09
WASO	126.8 (27.5)	132.7 (18.5)	0.73
Sleep efficiency (%)	70.2 (3.1)	74.0 (4.6)	0.24
Spindle density^∗^	5.5 (0.03)	5.8 (0.1)	0.001
Spindle amplitude (μV)	12.9 (0.05)	14.1 (0.05)	<0.001
Arousal index^∗∗^ stage N2	11.0 (4.0)	11.5 (2.9)	0.85
Arousal index stage N3	3.5 (1.0)	3.6 (1.1)	0.94
Arousal index total	13.6 (2.9)	12.8 (2.3)	0.84

All units are in minutes except if indicated otherwise. REM, rapid eye movement; WASO, wake after sleep onset. *Per minute of PLL activity, ^∗∗^number of arousals per hour.

### Acoustic Stimulation Increased SWA during ON Intervals

**FIGURE 4:**
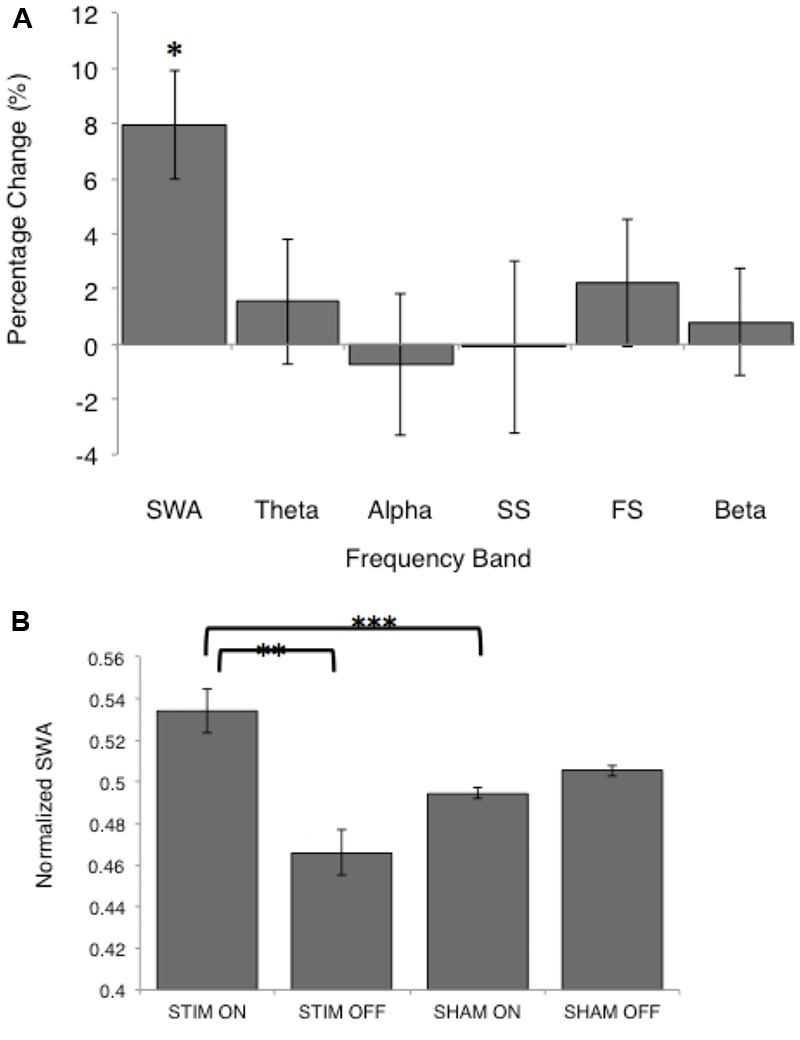
**Normalized spectral power for the STIM and SHAM periods. (A)** Percentage change from the SHAM ON to the STIM ON condition for SWA (>0.5–4 Hz), theta (>4–8 Hz), alpha (>8–12 Hz), slow spindle (SS, >11–13 Hz), fast spindle (FS, >13–15 Hz), and beta (>16–20 Hz) frequency bands. The change in SWA was significantly increased following STIM compared to SHAM (^∗^*p* = 0.006). **(B)** SWA for the STIM and SHAM, ON and OFF intervals. ^∗∗^*p* = 0.006, ^∗∗∗^*p* = 0.002 for paired *t*-tests.

There was no difference in average SWA during the entire NREM period for the two conditions [STIM: 121.7 (42.8) μV^2^/Hz, SHAM: 124.4 (41.8) μV^2^/Hz, *t*_12_= -0.27, *p* = 0.80]. Despite no net change in SWA, average SWA in the ON intervals was significantly higher than SWA in OFF intervals for the STIM night [STIM ON: 65.5 (11.0) μV^2^/Hz, STIM OFF: 54.1 (8.0) μV^2^/Hz, *t*_12_ = 2.6, *p* = 0.02]. There was no difference in SWA between ON and OFF intervals of the SHAM night [SHAM ON: 57.6 (7.6) μV^2^/Hz, SHAM OFF: 58.7 (7.5) μV^2^/Hz, *t*_12_ = 1.7, *p* = 0.12]. Normalizing the power spectra to the total power in ON and OFF intervals for the STIM and SHAM revealed an 8% increase in SWA in the STIM ON intervals compared to the SHAM ON intervals (*t*_12_ = 4.0, *p* = 0.002, **Figure 4A**). Whereas normalized SWA increased during STIM ON intervals compared to both STIM OFF (*p* = 0.006) and SHAM ON (*p* = 0.002), the difference in the SHAM ON and OFF intervals was not statistically significant (*p* = 0.10, **Figure 4B**). There were no significant differences between other frequency bands (**Figure 4A**, *p* > 0.05). However, theta power in the ON intervals of the STIM night was 1.6% higher than in the ON intervals of the SHAM night, but was not significant (*t*_12_ = 1.8, *p* = 0.09). Fast spindle power also showed a non-significant 2.2% increase in the STIM ON intervals compared to SHAM ON (*t*_12_ = 2.6, *p* = 0.14).

### Sustained SWA and Spindle Activity across ON Intervals

**FIGURE 5:**
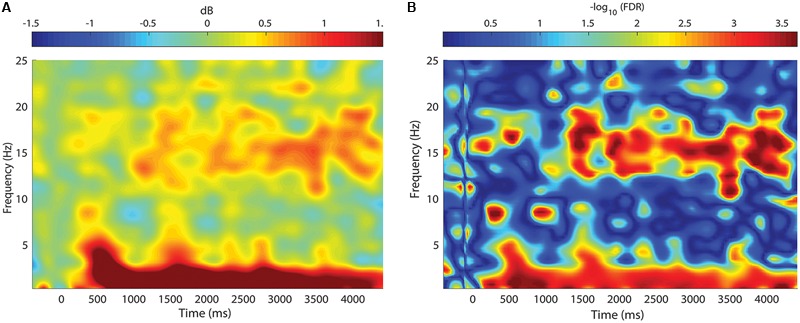
**Change in spectral power between STIM and SHAM conditions. (A)** Frequency by time plot for the STIM – SHAM ON intervals at Fpz, time locked to the onset of each five-pulse interval (time = 0). There was a robust increase in SWA, with smaller changes in theta activity beginning 500 ms after sound delivery. A modest increase in spindle activity was seen beginning at 1000 ms, with the greatest change at 3000 ms after sound delivery. **(B)** Significance of spectral power differences between the STIM ON and SHAM ON intervals. Color corresponds to the FDR-corrected *p*-values on a negative log scale, with dark blue corresponding to least significant (FDR = 1) and red corresponding to most significant (FDR = 0.0002).

Event-related spectral perturbations revealed time-varying changes in spectral content between the STIM and SHAM conditions (**Figure 5A**). Stimulation resulted in a robust increase in SWA activity, beginning ∼500 ms following the first pulse, with a modest increase in theta activity. There was a sustained increase in SWA for the entire 5000 ms epoch, with the largest increase at 600 ms. Smaller, but significant increases in spindle activity were seen 1000 ms following the first pulse. The largest increases in spindle activity were seen 3000–4000 ms following pulse delivery. Significant differences are depicted in **Figure 5B**.

### Acoustic Stimulation Enhanced Memory Consolidation

**FIGURE 6:**
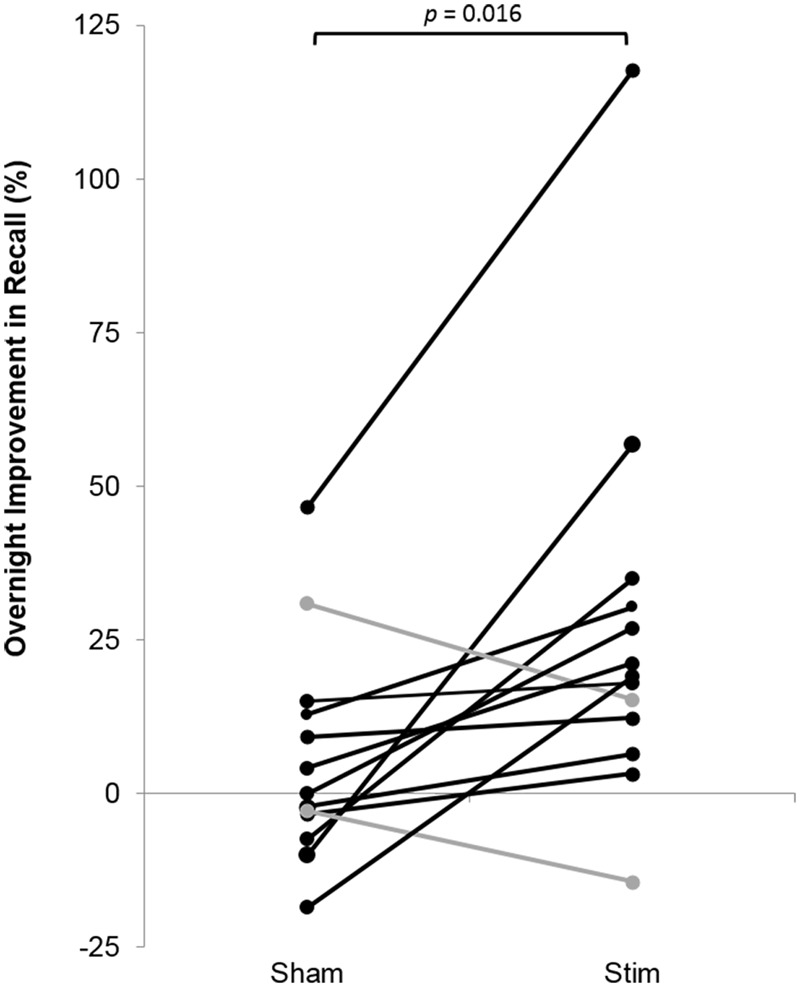
**Overnight percent improvement in recall for each subject for the STIM and SHAM conditions.** A positive value indicates memory improvement. Black indicates participants with a larger overnight improvement in recall in the STIM compared to the SHAM condition. Gray represents two participants who performed worse in the STIM compared to the SHAM condition. The increase in word pair recall following STIM was significantly higher than SHAM (*p* = 0.016).

Participants recalled a similar number of words prior to sleep on the STIM and SHAM evenings, (**Table 2**). The mean number recalled in the evening, collapsed across conditions, was 43.8 (18.5) words. Scores were generally higher in the morning, with a mean number of 50 (21.3) words recalled. Most importantly, recall increased by a mean of 9.2 (2.1) words following STIM and 3.1 (1.8) words following SHAM in the morning compared to the prior evening (*t*_12_ = 2.6, *p* = 0.02). We also compared the percent overnight improvement in recall from evening to morning as computed in each individual. This improvement averaged 26.8 (8.2)% in the STIM condition and 5.7 (4.5)% in the SHAM condition. Similarly, this measure showed a significantly greater improvement following a night of stimulation compared to SHAM (*t*_12_ = 2.8, *p* = 0.016). The change in word recall was highly consistent, and only two participants showed the opposite pattern (**Figure 6**). Meanwhile acoustic stimulation did not alter self-reported measures of sleep quality, mood, or alertness (Visual Analog Scale) between the STIM and SHAM nights (**Table 3**).

**Table 2:** Mean (SEM) number of word pairs recalled in the evening and the morning for the STIM and SHAM conditions.

	SHAM	STIM	*p*-value
**Number of word pairs recalled**			
Evening	44.8 (5.1)	42.9 (5.3)	0.40
Morning	47.8 (6.0)	52.1 (6.0)	0.22
Change (morning–evening)	3.1 (1.9)	9.2 (2.2)	0.02

*Mean change in number of words recalled from evening to morning.*

**Table 3:** Subjective sleep quality and global scores for the Visual Analog Scale for the STIM and SHAM nights.

	SHAM	STIM	*p-*value
**Karolinska Sleep Diary (KSD)**			
How did you sleep? (1 = very poorly, 5 = very well)	3.6 (0.23)	3.6 (0.20)	1
Did you feel refreshed after you arose this morning? (1 = not at all, 5 = completely)	4 (0.25)	3.6 (0.25)	0.27
Did you sleep soundly? (1 = very restless, 5 = very soundly)	3.5 (0.29)	3.6 (0.27)	0.58
Did you sleep throughout the time allotted for sleep? (1 = woke up much too early, 5 = slept through the night)	3.7 (0.24)	3.9 (0.32)	0.55
How easy was it for you to wake up? (1 = very easy, 5 = very difficult)	1.3 (0.16)	1.5 (0.20)	0.27
How easy was it for you to fall asleep? (1 = very easy, 5 = very difficult)	2.4 (0.34)	2.6 (0.27)	0.54
KSD Global Score	21.3 (0.85)	21.7 (0.86)	0.72
**Visual Analog Scale (VAS)**			
How Alert do you feel? (0 = not at all, 100 = very much)	80.3 (3.9)	77.2 (5.3)	0.38
How sleepy do you feel?	14 (4.6)	12.6 (5.3)	0.81
VAS Global Score	37.1 (0.91)	38.2 (1.2)	0.33

### Relative Change in SWA Predicted Improvement in Memory

**FIGURE 7:**
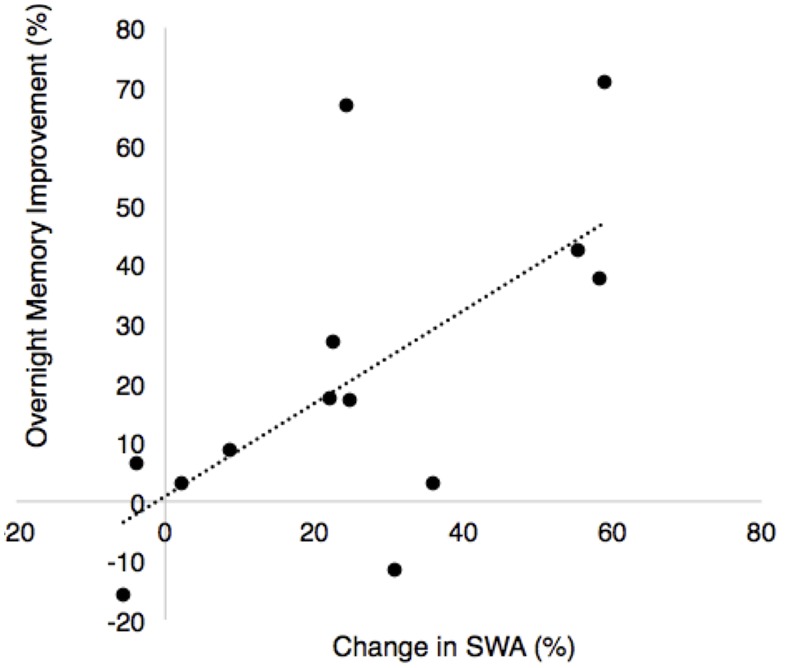
**Correlation between overnight percent improvement in recall between nights and the percent change in SWA for the ON and OFF intervals between nights (% change STIM – % change SHAM, *r* = 0.64, *p* = 0.018)**.

There was a significant positive association between the change in SWA (ON–OFF) and the stimulation effect on percent improvement in recall (*r* = 0.64, *p* = 0.018, **Figure 7**). There was no association between fast spindle activity and recall (*r* = -0.25, *p* = 0.41). Since spindle density was significantly different between the STIM and SHAM conditions we examined correlations between spindle density and recall performance for the STIM and SHAM condition separately. There were no significant correlations in spindle density for the STIM condition (*r* = 0.17, *p* = 0.53) or SHAM condition (*r* = -0.003, *p* = 0.95). There were no associations between total average SWA in NREM sleep and memory performance in either condition. Time spent in SWS was also not associated with an improvement in memory for either condition (STIM: *r* = 0.38, *p* = 0.2; SHAM: *r* = 0.07, *p* = 0.81).

### Relationships between Slow Wave Phase Targeting and Memory

**FIGURE 8:**
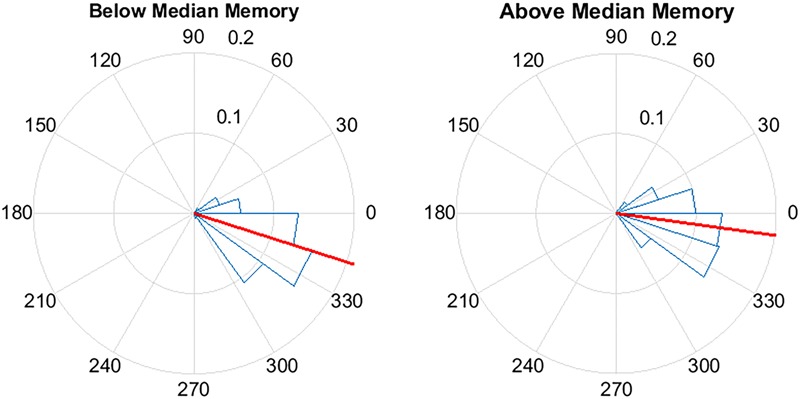
**Pulse delivery was significantly closer to the upstate (360/0°) in participants with above-median improvement in memory between the STIM and SHAM night.** Above-median (*n* = 7) memory performance was defined as ≥17% improvement in word recall and below-median (*n* = 6) improvement was defined as < 17% improvement. Circular y-axis indicates normalized counts. Red lines indicate mean vector, while blue triangles indicate bin spread (*p* < 0.0001).

To further explore the relationship between memory change and stimulation, for each participant we considered the phase of the slow wave at the time of stimulation. Across participants, there was a positive association between the mean phase of stimulation and percent recall improvement (*r* = 0.63, *p* = 0.02). As an additional way to visualize this effect, participants were divided into below-median (<17%, *n* = 6) and above-median (≥17%, *n* = 7) groups based on percent recall improvement between the STIM and SHAM conditions (**Figure 8**). The target phase of stimulation was 340 degrees, with the peak of the upstate represented at 360/0 degrees. Mean (SD) phase was 342.3 (21.9) degrees for the below-median group and 352.1 (23.2) degrees for the above-median group. Participants in the above-median group for differential word recall had a mean phase closer to the upstate compared to the below-median group (*p* < 0.0001).

## Discussion

Acoustic simulation that was phase-locked to sleep slow waves in older adults had systematic effects on sleep indices and performance on a declarative memory test. These results provide the first demonstration that acoustic stimulation alters SWA and can enhance word pair recall in older adults. These results converge with other findings in young adults indicating that acoustic stimulation during sleep is a promising tool for altering SWA and enhancing sleep-dependent memory consolidation (Ngo et al., 2013b; Ong et al., 2016). The present study showed that, despite their age-related decrease in the amount of SWS and SWA (Bliwise, 1993; Landolt et al., 1996), older adults can still gain from phased-locked acoustic stimulation, which can effectively enhance both sleep and memory consolidation.

The experimental procedure successfully targeted 20–30 degrees prior to the peak of the upstate of slow waves and elicited an increase in EEG amplitude. The feasibility of this method is encouraging given that older adults have smaller amplitude slow waves and less SWA than younger adults (Petit et al., 2004; Edwards et al., 2010). Despite maintaining the same slow wave criteria as in younger adults the PLL was able to track and target endogenous slow waves in an older population. In line with previous PLL studies in young adults (Ong et al., 2016; Santostasi et al., 2016) there was improved targeting for large amplitude slow waves. The advantage of using the PLL in the context of older adults is that it can be adapted to track and target any endogenous frequency within a designated target window (i.e., 0.5–4 Hz) and amplitude.

As observed in previous studies, acoustic stimulation did not result in an increase in the number of slow waves *per se* (Ngo et al., 2013a,b; Ong et al., 2016; Santostasi et al., 2016); rather, it led to an increase in amplitude and SWA during the period immediately following stimulation. The increase in amplitude following each pulse of the ON interval indicates a persistent EEG response to stimulation. The effect of auditory evoked responses on amplitude during sleep have been extensively studied, though primarily conducted in stage N2 sleep (Campbell and Bartoli, 1986; Colrain and Campbell, 2007), where naturally occurring slow oscillations are less frequent. Auditory evoked responses during N3 have been shown to be smaller in amplitude, have a sustained late negativity (Ujszászi and Halász, 1988; Bohlman-Nielsen et al., 1991), and are influenced by the phase of the slow oscillation (Riedner et al., 2011; Schabus et al., 2012). Findings from Ngo et al. (2013a) suggest phase-dependent stimuli lead to induction of the slow oscillation as opposed to an evoked response alone. However, the adaptive nature of the phase locked stimulation method used in this study does not allow for a clear separation of evoked and induced responses. When examining the power spectral density, while there was a selective increase in SWA seen during the STIM ON intervals compared to SHAM ON intervals there was no difference in total NREM SWA between nights. These data suggest that phase-locked acoustic stimulation results in a re-organization of SWA, rather than an overall increase in SWA.

Time-frequency analysis revealed that changes in theta and SWA appeared ∼500 ms after the first pulse and that spindle activity increased 1000 ms after the first pulse. Interestingly, similar to phase-locked methodology used in young adults (Ong et al., 2016), increases in spindle activity were sustained, lasting until the end of the STIM ON interval. Recently, Ngo et al. (2015) reported a rapid decline in spindle activity following multiple trains of acoustic pulses. The different observed responses likely speak to the ability of the PLL to lock pulses to the desired phase of the oscillation, as opposed to a fixed ISI as employed elsewhere (Ngo et al., 2015). With repeated stimuli, a fixed ISI may result in stimulating outside the desired phase and therefore a tapering of the physiological response. Alternatively, this may be due to delivering acoustic stimuli primarily during SWS in the present study, unlike other paradigms that also specifically included stage N2 in the stimulation period.

There was a robust 26% increase in word pairs recalled following overnight STIM compared to SHAM, which is in line with previous work by Ngo et al. (2013b). The overnight improvement may reflect a combination of at least three factors: memory consolidation over time during both wake and sleep, memory strengthening during both the recall and feedback portions of the evening test, and the effect of sleep. However, the finding that STIM greatly enhanced memory compared to SHAM can be attributed specifically to the benefit of memory processing during sleep.

For the above-median performing participants, the phase of pulse delivery was closer to the peak of the upstate. This finding contributes to the growing body of evidence that phase of acoustic stimulation may be important for sleep-based memory consolidation (Batterink et al., 2016). The ability of the PLL to track the endogenous slow wave and target the upstate may explain the effectiveness of stimulation as there is evidence that the brain is more responsive during the upstate of the slow oscillation (Schabus et al., 2012). Ngo et al. (2013b) observed that stimulating out of phase with slow oscillations resulted in no change in SWA or memory, suggesting that the precise timing of stimulation is important. More recent findings support the hypothesis that quasi-phase dependent stimuli that enhance only SWA, but not fast spindle activity do not lead to memory consolidation (Weigenand et al., 2016). If, indeed, phase of stimulation matters, the PLL algorithm that can target specified phases of the EEG derived waveforms represents a novel method that can be adapted in a variety of settings and different clinical populations to enhance learning and memory.

The mechanism by which memory consolidation occurs during sleep is complex and perhaps even less understood in older adults. Thus far, studies in older populations have had mixed results: some groups have found positive associations between percentage of time spent in SWS or amount of SWA and paired-associate recall (Backhaus et al., 2007; Westerberg et al., 2012), while others have not (Scullin, 2013). Part of the complexity may stem from age-dependent structural and functional alterations to the frontal cortex (Kalpouzos et al., 2009) that have shown to mediate the link between SWA and memory in older populations (Mander et al., 2013b). Meanwhile, reduced prefrontal fast spindle activity has been associated with reduced next day hippocampal function and learning ability in older adults (Mander et al., 2013a). Together, these findings suggest that the hippocampal-neocortical dialog, including both slow oscillations and spindles, may continue to be important for memory in old age.

In the present study, the association between the percentage change in the ON and OFF intervals and memory suggests a transient re-organization of SWA may be the critical component to memory consolidation during sleep. This association is intriguing because participants spent less time in SWS during the STIM night than during the SHAM night without increased arousals, yet improvement in declarative memory was robust. Therefore, transient re-organization, rather than overall SWS or SWA, may have contributed to the memory enhancement. Acoustic stimulation may drive the cortical slow oscillation (Steriade, 2006) to synchronize neuronal up and down states, facilitating hippocampal-to-neocortical information transfer and persistence of long term memories in the cortex (Sirota et al., 2003; Marshall et al., 2006). Despite increases in spindle activity following stimulation, we found no significant associations with memory as seen in other studies in young adults (Ngo et al., 2013b). This discrepancy can potentially be explained by duration of stimulation (SWS only vs. stage N2 and SWS), mechanistic differences in the relationship between spindle activity and memory consolidation in older adults, or simply lack of statistical power. Nevertheless, it is possible that acoustic stimulation ultimately serves as a mechanism to assist in making sleep more efficient.

The small sample size in the present study has limited our ability to detect associations of sleep macrostructure, such as time in SWS or spindle density, with memory outcomes. Timing of acoustic pulse delivery was estimated using internally generated computer delays instead of an external trigger monitor. While we compensated for this delay in stimulus delivery, there may be a small additional hardware delay from computer to headphones that is not accounted. In addition, although individuals with hearing impairments were excluded, an objective measure of hearing such as with brainstem acoustic evoked responses was not obtained. Thus, given the older study population, some of the variability in effectiveness of the stimulation may be due to differences in age-related hearing loss.

These results pose the need for more investigation into the mechanisms by which memory processing occurs during sleep. While there are hypotheses on acoustic stimulation activating “bottom-up” processes contributing to neuronal synchronization (Bellesi et al., 2014), it is not known how the presence of sound might modulate endogenous oscillations and spindles that alter memory. Additionally, while relationships of spindle activity to memory consolidation have been proposed in younger adults, such mechanisms remain elusive in older populations (Harand et al., 2012). While this study did not aim to test varying slow wave criteria for stimulation in an older population, it may be possible that other settings elicit even stronger responses. Given that the same parameters were used as in younger populations, future studies should aim to test and further optimize parameters for different populations. Finally, more research is needed to understand the effects of repeated nights of acoustic stimulation on brain physiology and memory, particularly in the older adult population. Age-related changes in sleep and memory related brain regions (Mander et al., 2015) may mean that older adults, particularly those at high risk for cognitive impairment stand to gain the most from such an intervention. While the present study examines a single night of stimulation, the current methodology allows for flexible at-home use. Therefore, future studies should focus on repeated use, potentially over the course of several weeks, as a potentially useful approach to improve both sleep and memory for high-risk populations. Acoustic stimulation during sleep has the potential to be a practical and safe tool for enhancing the sleep-dependent effects on memory in healthy older adults, as well as those with cognitive impairment.

## Author Contributions

NP, GS, and RM collected and analyzed the data. RB provided statistical analysis. GS, RM, SW, KP, and PZ designed the study. NP, GS, RM, RB, SW, KP, and PZ wrote the paper.

## Conflict of Interest Statement

GS, PZ, and KP have filed a provisional patent for the phase-locking technique used in this manuscript with the United States Patent and Trademark Office (U.S. Patent Application No. 62/038,700).

The other authors declare that the research was conducted in the absence of any commercial or financial relationships that could be construed as a potential conflict of interest.
